# Functional Autonomy Affects Elderly Spatial Perception in Body-Centered Coordinates

**DOI:** 10.1155/2020/5694790

**Published:** 2020-02-20

**Authors:** Giorgia Committeri, Valentina Sebastiani, Francesco de Pasquale, Massimiliano Stocchi, Chiara Fini

**Affiliations:** ^1^Department of Neuroscience Imaging and Clinical Sciences, and ITAB, University G. d'Annunzio of Chieti-Pescara, Chieti Scalo, Italy; ^2^Faculty of Veterinary Medicine, University of Teramo, Piano D'Accio, Teramo, Italy; ^3^Igea, Centro Promozione Salute, Pescara, Italy; ^4^Department of Dynamic and Clinical Psychology, State University of Rome “La Sapienza”, Rome, Italy

## Abstract

According to the action-specific theory of perception, a person's dynamic ability to act in the environment affects her/his spatial perception. Empirical evidence shows that the elderly perceive distances as farther compared with younger adults and that the harder the ground surface to walk, the farther the perceived distance. Such results suggest a general perceptual readaptation promoted by the aging process that is fine-tuned with the decline of the motor resources. However, it is still unknown whether the elderly space perception is affected by interindividual differences in their functional autonomy (FA) and whether the decline of motor resources affects spatial categorization only when distances are judged with reference to the observer's own body or also when they are judged with reference to the body of another agent present in the scene. To this aim, a sample of elderly adults with preserved cognitive functions but different levels of FA, measured through the Instrumental Activity of Daily Living (IADL) scale, were enrolled and tested on the extrapersonal space categorization task. This task requires judging the position of a target as “Near” or “Far” with respect to different reference frames (RFs): centered on the observer's body (Self RF) or centered on external elements, like another body (Other RF) or an object (Object RF). Results indicated that the higher the level of FA, the wider the space categorized as “Near” when adopting as reference frame our own body or the body of another agent in the scene, but not a static object. In conclusion, the individual functional autonomy of elderly individuals, which is strongly influenced by motor resources and efficiency, modulates how the surrounding space is represented, but only when the distance judgment implies an agent body, thus providing new relevant data for recent embodied cognition models of aging.

## 1. Introduction

According to the action-specific theory of perception [1], a person's dynamic ability to act in the environment, which is largely determined by her/his body (size, control and coordination, and energetic potential), affects her/his spatial perception. These effects are suggested to be potentially adaptive for planning future actions based on the perceiver's abilities rather than on behaviorally irrelevant metrics [1].

Within the extrapersonal space (i.e., space beyond reaching distance: [2]), pioneering research by [3], for example, demonstrated that hills are judged as steeper when people are fatigued or carry a heavy backpack. Similarly, people who are out of shape and have poor physical fitness levels or a low amount of available metabolic energy judge the hills to be steeper compared with fit individuals [4, 5]. Still, increased anticipated effort for walking leads to increased judgment of distances [6, 7]. Interestingly, also the categorization (near/far) of spatial distance is affected by the actor's action possibility. For example, we previously observed in young adults that targets are perceived nearer with reference to another person's body with movement opportunities (an avatar) as compared with a person without them (a wooden dummy) or an inanimate static object [8–10]. Notably, by inducing the idea that the wooden dummy is a biological agent (like Pinocchio) people who were more transported into Pinocchio's story made similar spatial categorizations using the wooden dummy and the avatar with real movement possibilities [11].

Given that older adults typically manifest concurrent physical and cognitive changes [12], they represent a privileged occasion for testing action-perception links as well as a natural fit for the embodied cognition theoretical framework (see [13], for review). Not surprisingly, elderly people judge the hills as steeper compared with younger adults [3]. More recently, it has also been shown that older adults perceive distances as farther compared to young adults and that their verbal estimates of target distance are sensitive to the floor surface, with greater perceived distances on a slippery one, due to greater anticipated walking effort and increased risk of falling [14].

However, the great majority of studies focused on older adults as a group, largely neglecting interindividual differences in relevant embodiment factors [13]. One of these factors, still neglected, is the level of functional autonomy in daily life, which is strongly associated with physical performance [15] and is crucial when considering the interaction between an individual and her/his surrounding environment. At equal age and preserved general cognitive level, there are older people maintaining their autonomy by continuing, for example, to go out for purchases, while physical and/or familial conditions force some others to a very early assistance for daily activities. In the latter case, life in assisted living facilities is frequent, where transfers and interactions with the surrounding space are strongly limited.

A still open question, therefore, is whether interindividual differences in functional autonomy and related motor resources/efficiency within the elderly population affect distance perception. Another unexplored question is whether the decline of motor resources affects spatial perception only when distances are judged with reference to the observer's own body as previously observed [14] or also when they are judged with reference to the body of another agent present in the scene, given that we automatically encode his/her action's possibility [8, 16].

In order to clarify these issues, here we compared cognitively preserved elderly participants with different levels of functional autonomy, measured through the Instrumental Activities of Daily Living (IADL, [17]) scale, on the extrapersonal space categorization task (e.g., [16]). This task allows to evaluate the portion of extrapersonal space judged as near (i.e., accessible) by using different reference frames (RFs): centered on the observer's body (egocentric or Self RF) or centered on external elements (i.e., allocentric) like the body of another agent present in the scene (Other RF) or an inanimate object (Object RF). Following the results of previous studies in the field of extrapersonal space perception, we expected an influence of the functional autonomy level on distance categorization only during body-centered (own body or another body) judgments. Specifically, participants with higher autonomy levels were expected to categorize as “Near” a higher portion of space only when using a Self or Other RF (versus Object RF).

## 2. Materials and Methods

### 2.1. Sample and Neuropsychological Assessment

Twenty-two elderly individuals (13 females: mean age = 83.3 years and range = 74–93 years; mean education = 9.2 years and range = 4–18 years) were enrolled. All subjects were right-handed, had normal or corrected-to-normal visual acuity, and were naïve as to the purposes of the experiment.

Participants provided written informed consent prior to enrolment in the study, which conformed with ethical guidelines of the 1975 Declaration of Helsinki and was approved by institutional research board.

Participants were recruited among community dwellers, those attending a social center for elderly people and from an assisted living facility. No subjects were excluded due to medical or psychiatric history. The general cognitive level was assessed through the Italian version of the Mini Mental State Examination (MMSE, [18]), widely used in the elderly population to evaluate the presence of cognitive impairment and including tests of orientation, memory, attention, language, and visuospatial abilities. We excluded participants scoring below the cut-off that corresponds to the adjusted score of 22 (score range 22.2–30; mean: 26.8; s.d.: 2.2).

The level of functional autonomy was evaluated through the Instrumental Activities of Daily Living (IADL, [17]) scale, which assesses the autonomy in basic daily activities beyond personal care, like routine multistep functional tasks requiring high-level competencies and interactions with the surrounding environment and with others (e.g., use the telephone and means of transportation; make purchases). Moreover, elderly people spend half of their time engaged in IADL [19].

Since the IADL score refers to different scales in the two genders, from 1 to 5 for males and from 1 to 8 for females, a single percentage score has been generated (mean = 64.2%; range = 12.5%–100%). Some of the participants regularly used walking aids (13.6%).

### 2.2. Experimental Stimuli, Task, and Procedures

We administered the extrapersonal space categorization task [8, 16] to all the participants.

**Figure 1:**
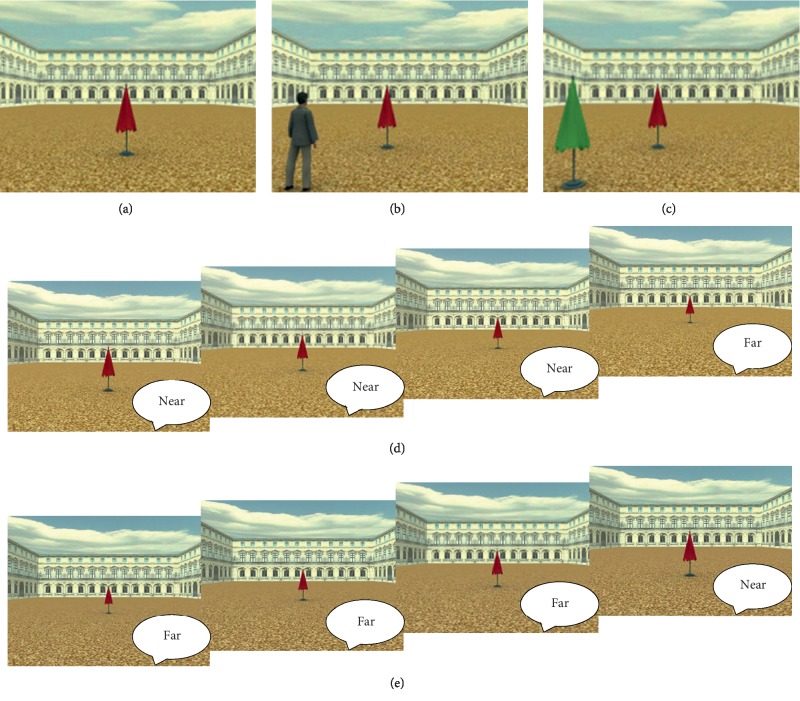
Exemplar stimuli and series used in the experiment. RF = reference frame. (a) Egocentric Self RF; (b) allocentric Other RF; (c) allocentric Object RF. (d) Hypothetical example of an ascending series for the Self RF. From left to right, pictures are presented until the participant changes his/her judgment from “Near” to “Far”. Picture shows distances 10–16 m in steps of 2 m but the ascending series started always at 2 m. (e) Hypothetical example of a descending series for the Self RF. As for (d), pictures are presented until the participant changes his/her judgment from “Far” to “Near”. Picture shows distances 16–10 m in steps of 2 m but the descending series started always at 54 m.

Stimuli included a 3D scene created by means of a virtual reality software (3D Studio Max 4.2, Autodesk, Discreet). The scene was a 3D environment, representing a square arena palace (Figure 1). In the first set of stimuli (Figure 1(a)), a red target umbrella was present on the scene, along a central vector aligned to the central camera (Self reference frame or RF). In the second set of stimuli (Figure 1(b)), a virtual man or avatar was located 45° to the right (left) of the central camera representing the participant's perspective, and the target red umbrella was located along a central vector aligned with the RF (Other RF). The third set of stimuli (Figure 1(c)) was identical to the second one, except for the presence of a green beach umbrella (Object RF) instead of the avatar. The first set of stimuli is the same as the one used in [16], whereas the second and third are the same as the ones used in [8]. Stimuli were presented on a full screen 17′ computer display placed at 57 cm from the subject.

We administered the stimuli through the limit method. Each experimental series started with a white fixation cross on a black background (2.500 ms) and consisted of 27 potential trials in which the red beach umbrella was located at 27 different distances (from 2 m to 54 m) from the reference frame (RF). Each trial lasted until the response was done and was followed by a white fixation cross (1.5 × 1.5 cm) on a black background for 2.500 ms. Subjects were asked to judge whether the red beach umbrella was “Near” or “Far” from the three different RFs, by verbally expressing the “Near/Far” judgment. We opted for this method of registering the responses, to overcome the absence of familiarity with the computer by the elderly people. In ascending series, the red umbrella was progressively moved away from the RF until the participants provided the “Far” judgment (Figure 1(d)). In descending series, the red umbrella was progressively moved closer to the RF until the participants provided the “Near” judgment (Figure 1(e)). Then the following series started. The points where participants expressed a transition from “Far” to “Near” (descending series) and from “Near” to “Far” (ascending series) were averaged for each subject in order to calculate a Judgment Transition Threshold (JTT) for each RF. Higher JTT values show a categorization of space as “Near” at longer target distance compared to lower JTT values. In other words, the higher the JTT, the broader the space categorized as “Near”. Each series was repeated 4 times for each RF. Each subject was thus submitted to 24 randomized experimental series (3 RFs: Self, Other, and Object x 8 series type: 4 ascending and 4 descending). The presentation of the stimuli and the recording of the participant's responses were controlled by the software E-prime 1.2.

Before starting, the participants were instructed by two experimenters about the task: one of them verbally explained the instructions, while the other executed the task to directly show it to the participants. Moreover, all participants were submitted to a training. Only when they demonstrated to have clearly understood the task and their responses were consistent, the experimental session was started.

### 2.3. Data Analysis

Trials below or above two standard deviations from the average threshold in the single participant were treated as outliers and excluded from the analysis (percentage of excluded trials in the total sample: 1.51%).

In our analyses, we considered models of increasing complexity. First, we tested the strength of linear association between JTTs (corresponding to different RFs: Self, Other, and Object) and IADL percentage values, age, and MMSE corrected score by means of a robust correlation analysis (Pearson's skipped correlation coefficient) that ignores detected outliers by taking into account the overall structure of the data [20]. However, this corresponds to modelling all the possible pairs of JTT associations independently, thus neglecting the effect of eventual joint covariations of these variables on JTT. To overcome this limitation, we then adopted a multiple linear regression approach by considering all the possible combinations of all observed variables and their interaction on JTTs (with different RFs: Self, Other, and Object).

## 3. Results

As reported in Table 1, a significant positive skipped Pearson's correlation was obtained between the IADL percentage score and the spatial JTT with both the Self RF and the Other RF. Crucially, the correlation with the Object RF was not significant and neither the Self JTT nor the Other JTT showed a significant correlation with age and MMSE corrected score. As previously reported in literature [21], IADL and MMSE scores were positively correlated.

**Figure 2:**
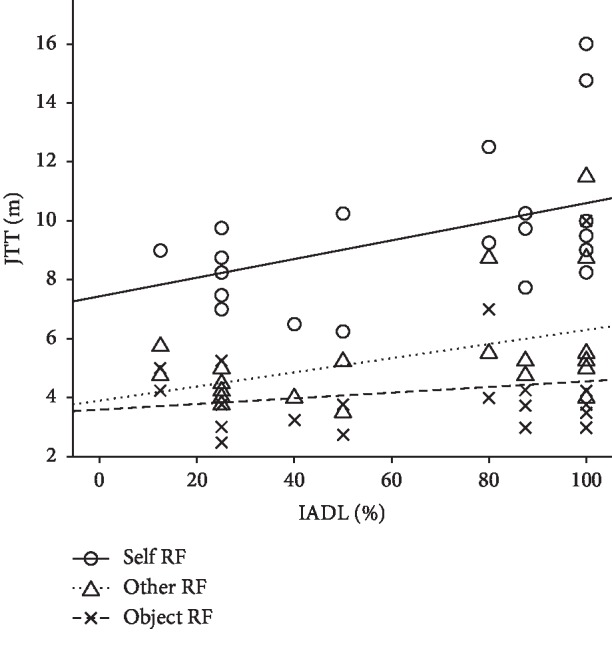
Regression analyses results. Scatter plot and corresponding regression lines for the relationship between the dependent variable mean Judgment Transition Threshold (JTT) referring to the three different RFs (Self, Other, and Object) and the independent variable IADL (Instrumental Activities of Daily Living) percentage scores.

Due to such observed multicollinearity, in our subsequent regression analyses the predictor MMSE was excluded. Among all the possible considered scenarios (corresponding to all the combinations of IADL, age, and their interactions), only the model including IADL resulted statistically significantly in predicting spatial JTT, in both Self RF (*F*_(1,20)_ = 5.33; *p* = 0.03; *R*^2^ = 0.21) and Other RF (*F*_(1,20)_ = 4.74; *p* = 0.04; *R*^2^ = 0.44) (Figure 2). The details of the regression analysis of all the considered models are reported in Table 2 [22].

## 4. Discussion

Given their concurrent physical and cognitive changes [12], the elderly represent a privileged occasion for testing action-perception links as well as a natural fit for the embodied cognition theoretical framework [13]. In the field of spatial cognition, much work has been devoted to the effects of aging on spatial memory and navigation (see [23]), with particular attention to the changes of egocentric and allocentric spatial representations/reference frames. Impairments of allocentric coordinates have been often documented (e.g., [24–27]), but also deficits in learning spatial environments when operating through an embodied and first-person perspective, as well as a reduced efficiency in the multisensory integration of the bodily signals (kinesthetic, tactile, proprioceptive, and interoceptive), with overreliance on visual information (for review, see [13]).

Fewer studies investigated spatial perception and distance judgments in the extrapersonal space, finding that older people, compared with younger adults, estimate hills as steeper [3] and distances as farther, depending on the requested level of motor control [14]. In the current study, instead of comparing older with younger adults, we considered the interindividual differences in functional autonomy within the elderly sample in order to study action-perception links within the embodied cognition framework. In the elderly, indeed, the speed of travel, the time to get up from the sitting position, and the ability to walk tandem (one foot in front of the other) are independent predictors of the ability to perform Instrumental Activities of Daily Living [15]. Crucially, these routine multistep functional tasks (e.g., use the telephone and means of transportation; make purchases) require interactions with the surrounding environment and with others. Moreover, given that we automatically encode the action's possibility of another body present in the scene during distance categorization (near-far judgments) [8, 16], in the present study, we also investigated whether the decline in functional autonomy affects spatial perception only when distances are judged with reference to the elderly own body [14] or also when they are judged with reference to the body of another agent present in the scene with respect to an object without motion potentialities.

In line with our expectations, correlational and regression analyses converged in showing that the level of functional autonomy, and not the age by itself, predicts how large is represented the “Near” space with respect to body-centered reference frames. Specifically, the higher the functional autonomy, the wider the extension of the portion of extrapersonal space judged as “Near” during both egocentric body-centered (Self RF) and allocentric body-centered (Other RF) distance judgments, but not during allocentric object-centered one (Object RF).

For what concerns the main result, subject-specific level of functional autonomy appears related to distance categorization in egocentric coordinates: the more the elderly people are dynamic and able to manage their daily activities, the more they judge and categorize the surrounding space as near to them, thus being accessible. Age does not exert an all-or-none effect in egocentric spatial representation, but this is modulated by an individual's ability to autonomously function in the community setting, mainly based on her/his physiological and motor resources. The general cognitive level measured through the Mini Mental State Examination (MMSE, [18]), indeed, is not sufficient to explain the observed differences. The MMSE score was normal in all participants by design and it was not correlated to the extension of the measured spatial thresholds. The dissociation between the effects of functional autonomy on egocentric but not allocentric object-centered judgments is in line with their dissociated neural substrates, with the egocentric representations selectively tapping on dorsal stream regions [28–30] devoted to action [31].

Notably, our findings indicate that distance judgments were affected by the level of functional autonomy also when using an allocentric but (Other) body-centered reference frame. Our previous data on young adults showed that targets are perceived nearer with reference to another person's body with movement opportunities as compared with a person without them or an inanimate static object [8]. A human body is therefore a “special allocentric reference frame” [8] that would induce an automatic perspective taking [32, 33], probably sustained by a self-projection motor simulation mechanism [9]. The present data bring further support to this idea, because the effects of another person's action (specifically, walking) potentialities on the observer's judgments appear to be actually modulated by the observer's own abilities as if she or he was in the other person's situation. In line with this result, Witt et al. [34] have put in evidence that when people with better or worse ball-blocking ability than their partner's abilities are asked to judge the speed in a ball-blocking paradigm observing the partner's performance, they still continue to filter the speed perception by their own abilities. This is consistent with action-specific accounts of perception [1, 4], which assert that the world is perceived in terms of the perceiver's ability to perform the intended action. Interestingly, a very recent study on spatial text processing showed that also time estimations, but not distance estimations, are affected by the age of the participants and that of the characters [35], probably because, in line with the embodied cognition approach, the elderly create a mental representation of the character's action potential that stems on the spatial reenactment of their own sensorimotor experience. Future research might manipulate also the age of the Other body used as RF during perceptual distance judgments, in order to clarify the relationship between own- and other-body characteristics within the embodiment process. Moreover, further studies employing more similar procedures “are needed to see whether spatial and temporal attributes are similarly or differently sensitive to the body's physical characteristics” [35].

Taken together, the present data speak in favor of embodiment in older adults. The malleability of both body-centered thresholds suggests their possible use as a proxy of an individual's involvement within the surrounding space. Since such a paradigm is easy to be administered, it might represent a useful instrument to measure “spatial involvement” and, in general, distance categorization with respect to different frames of reference in clinical populations. A space representation that maximizes the level of exploration/appropriation of the surrounding environment should indeed be actively preserved and/or empowered.

Before concluding, however, we have to acknowledge a limitation of the present study, consisting in the small sample size; therefore the present data must be considered as preliminary and future research will have to examine this issue on a wider number of participants, possibly also acquiring motor and physiological variables in order to better clarify the mechanisms underlying the observed effects.

## 5. Conclusions

In conclusion, the individual differences in motor resources/efficiency, as reflected by functional autonomy in the Instrumental Activities of Daily Living, predict the amount of subjective near extrapersonal space of elderly people whenever distance judgments are referenced to an agent body, thus bringing support to embodied theories of spatial cognition and enriching available models in the recent field of geriatric embodied cognition [13].

## Figures and Tables

**Table 1:** Skipped correlations.

		IADL	AGE	MMSE	Self RF	Other RF	Object RF
IADL	Pearson's *r*	—	−0.185	0.557	0.459	0.438	0.207
	*p* value	—	0.204	0.003^*∗∗*^	0.016^*∗*^	0.020^*∗*^	0.178
	95% CIs	—	−0.582	0.170	0.242	0.206	−0.262
		—	0.195	0.797	0.663	0.649	0.495
AGE	Pearson's *r*		—	−0.666	0.191	0.011	0.183
	*p* value		—	0.384	0.198	0.484	0.207
	95% CIs		—	−0.416	−0.238	−0.451	−0.288
			—	0.316	0.562	0.404	0.559
MMSE	Pearson's *r*			—	0.175	−0.053	−0.281
	*p* value			—	0.219	0.408	0.104
	95% CIs			—	−0.254	−0.501	−0.769
				—	0.559	0.397	0.232
Self RF	Pearson's *r*				—	0.729	0.467
	*p* value				—	0.000^*∗∗*^	0.015^*∗*^
	95% CIs				—	0.481	0.015
					—	0.877	0.736
Other RF	Pearson's *r*					—	0.492
	*p* value					—	0.010^*∗*^
	95% CIs					—	0.075
						—	0.763
Object RF	Pearson's r						—
	*p* value						—
	95% CIs						—
							—

^*∗*^ *p* < 0.05; ^*∗∗*^*p* < 0.01.

**Table 2:** Regression analyses details.

Model (*x*)	Self RF (*y*)				Other RF (*y*)				Object RF (*y*)			
	B	SE_B_	95% CI		B	SE_B_	95% CI		B	SE_B_	95% CI	
IADL	0.031	0.014	0.003	0.060	0.024	0.011	0.001	0.047	0.010	0.010	−0.012	0.031
	*R * ^2^ = 0.210; *F* = 5.329; *p*=0.032^*∗*^				*R * ^2^ = 0.192; *F* = 4.745; *p*=0.042^*∗*^				*R * ^2^ = 0.043; *F* = 0.893; *p*=0.356			

Age	0.096	0.101	−0.114	0.306	0.051	0.082	−0.120	0.221	0.077	0.069	−0.067	0.221
	*R * ^2^ = 0.043; *F* = 0.901; *p*=0.354				*R * ^2^ = 0.019; *F* = 0.384; *p*=0.543				*R * ^2^ = 0.058; *F* = 1.234; *p*=0.280			

IADL	−0.008	0.133	−0.286	0.271	0.059	0.110	−0.173	0.291	0.071	0.102	−0.144	0.285
Age	0.132	0.095	−0.068	0.332	0.089	0.079	−0.077	0.256	0.103	0.073	−0.051	0.256
IADL *∗* age	0.001	0.005	−0.008	0.011	−0.001	0.004	−0.009	0.007	−0.002	0.004	−0.009	0.005
	*R * ^2^ = 0.303; *F* = 2.611; *p*=0.083				*R * ^2^ = 0.245; *F* = 1.946; *p*=0.158				*R * ^2^ = 0.139; *F* = 0.972; *p*=0.428			

^*∗*^ *p* < 0.05; B = unstandardized regression coefficient; SE_B_ = standard error of the coefficient; CI =  confidence intervals.

## Data Availability

The dataset used and analyzed during the current study are available from the corresponding author upon reasonable request.
